# Microscopic analysis of the digestive and reproductive tracts of male *Silpha obscura* (Coleoptera: Silphidae)

**DOI:** 10.1093/aesa/saae042

**Published:** 2024-12-09

**Authors:** Michaela Urbanová, Ramona Babosová, Vladimír Langraf, Kornélia Petrovičová, Nurcan Özyurt Koçakoğlu, Martin Morovič

**Affiliations:** Faculty of Natural Sciences and Informatics, Constantine the Philosopher University in Nitra, Nitra, Slovakia; Faculty of Public Health, Department of Toxicology, Slovak Medical University in Bratislava, Bratislava, Slovakia; Faculty of Natural Sciences and Informatics, Constantine the Philosopher University in Nitra, Nitra, Slovakia; Faculty of Natural Sciences and Informatics, Constantine the Philosopher University in Nitra, Nitra, Slovakia; Faculty of Agrobiology and Food Resources, Slovak University of Agriculture in Nitra, Nitra, Slovakia; Science Faculty, Department of Biology Yenimahalle, Gazi University, Ankara, Turkey; Faculty of Natural Sciences and Informatics, Constantine the Philosopher University in Nitra, Nitra, Slovakia

**Keywords:** *Silpha obscura*, histology, morphology, digestive tract, reproductive tract

## Abstract

*Silpha obscura* Linnaeus, 1758 (Coleoptera: Silphidae) is an omnivorous beetle species known for damaging agricultural crops, making it an important target for pest control strategies. Despite its impact, no studies have yet examined the anatomical and histological structures of the male digestive and reproductive tracts of *S. obscura*. Therefore, this study aimed to describe these structures in detail, which could provide insights into potential control methods. Twenty individuals were collected and histologically processed, with observations made using light microscopy. The digestive tract of *S. obscura* comprises 3 parts: a short foregut, a relatively long and wide midgut characterized by folds and numerous crypts of the blind intestine, and a narrow and elongated hindgut, which expands into the rectum. Malpighian tubules attach at the beginning of the hindgut, which continues with the ileum, colon, and rectum and ends at the anus. The male reproductive tract includes a pair of testes, a pair of efferent ducts (vas efferentia), a pair of deferent ducts (vas deferentia), a pair of seminal vesicles, 2 pairs of accessory glands, and the ductus ejaculatorius, which extends into the ejaculatorius bulbus. The reproductive tract terminates with the aedeagus. The testes consist of 2 lobes, each containing numerous follicles where spermatogenesis and spermiogenesis occur. This study provides detailed insights into the anatomy and histology of the digestive and male reproductive tracts of *Silpha obscura*. These findings may lead to new strategies for controlling this agricultural pest. Understanding these structures is crucial for further research.

## Graphical Abstract

**Graphical Abstract:**
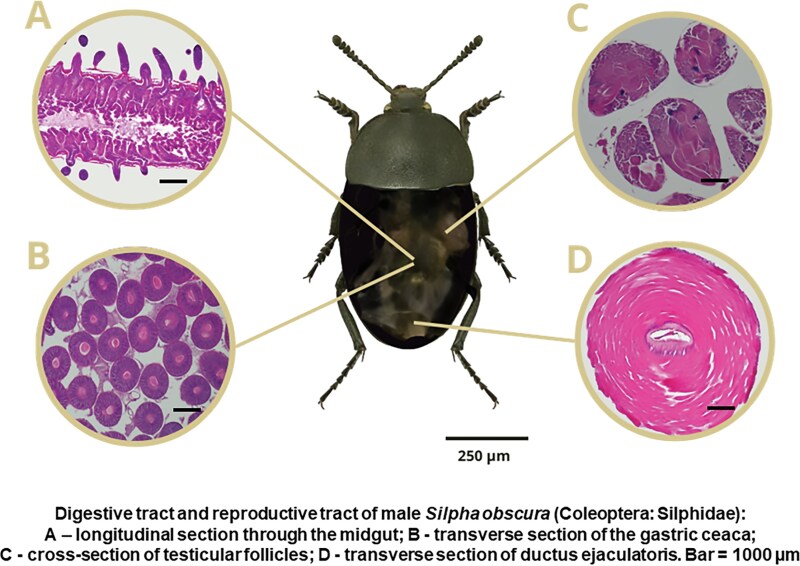

## Introduction

Silphidae is a small group of insects comprising 186 species, divided into 15 genera and further categorized into 2 subfamilies (Park and Moon 2020, Toni et al. 2022). Despite their relatively small number, Silphidae are distributed worldwide. The center of their distribution, with the most diverse genera, is the Palearctic and Nearctic regions (Dekeirsscheiter et al. 2011).

The majority of Silphidae species are necrophagous, but they are often omnivorous, causing damage to agricultural crops such as sugar beets, corn, and wheat (Çiftçi et al. 2018, Park and Moon 2020). However, it is their unique ability to locate carrion within a few hours of its death and colonize it, similar to flies, that makes certain Silphidae species potentially valuable for forensic investigations (Dekeirsscheiter et al. 2011, Park and Moon 2020, Trumbo and Newton 2022). This potential opens up exciting new avenues in the field of forensic entomology.

From a knowledge perspective, *Silpha obscura* is considered a less-studied beetle species not only in the territories of Slovakia and the Czech Republic but also globally.

The most prominent internal organ of Coleoptera is its digestive tract, the structure of which varies significantly depending on diverse feeding habits (Özyurt Koçakoğlu et al. 2021a, 2022). However, certain insights suggest that intestinal characteristics may be more correlated with phylogeny than with feeding habits (Mehrabadi et al. 2012, Toni et al. 2022). The digestive tract is a single long epithelial canal, distinctly separated by different structures and cell types, into 3 main parts: foregut, midgut, and hindgut.

The foregut is straight and tubular, consisting of the oral cavity, pharynx, esophagus, and proventriculus. The midgut, which is the primary site for macronutrient breakdown and absorption, includes the stomach and the gastric caeca appendages. It terminates at the Malpighian tubules, which represent the most active region of the digestive tract. The hindgut begins at the pyloric valve, comprising the ileum, colon, and rectum and terminates at the anus. Coleoptera are the most diverse group of insects, leading to potential variations in the digestive tract (Özyurt Koçakoğlu et al. 2021a, 2021b, 2022, Toni et al. 2022).

The internal morphology of beetles is commonly used for taxonomic classification of species. The reproductive tract is no exception, exhibiting significant morphological variability based on the vast diversity of insects (Erbey et al. 2021, Özyurt Koçakoğlu et al. 2023). The morphology of the male reproductive tract in Coleoptera consists of a pair of testes, a pair of efferent ducts, a pair of deferent ducts, a pair of accessory reproductive glands, and the ejaculatory duct. The reproductive tract of *S*. *obscura* is similar to that of other Coleoptera species belonging to the suborder Polyphaga. Differences in the reproductive system mainly involve the number and morphology of reproductive glands and the characteristics of the testes (Wu et al. 2017, Özyurt Koçakoğlu et al. 2021c, Özyurt Koçakoğlu and Candan 2022).

Currently, the available information on *S*. *obscura* is limited to its global distribution, with no research literature on the anatomy and histology of its digestive and reproductive tracts. Recognizing this gap, we have undertaken a comprehensive examination of the digestive and male reproductive systems of *S*. *obscura*. This study will provide a foundation for further research on the morphology of the digestive and reproductive systems and indirectly contribute to protecting against agricultural pests, including the analyzed species *Silpha obscura*.

## Materials and Methods

### Materials

Adult males of the species *S*. *obscura* (*n* = 20) were used. They were collected using pitfall traps without bait in an agricultural area on wheat fields, specifically at 48°17ʹ01.0″N 18°06ʹ56.6″E near Nitra, Slovakia, during the period from May to July 2022. The individuals were transported to the laboratory, where they were euthanized using chloroform via inhalation. Dissection was performed under a stereomicroscope (STM 712), and prepared samples were fixed for 24 h in Davidson’s solution (300 ml distilled water, 200 ml formaldehyde p.a. (Diapath, Italy), 100 ml concentrated acetic acid, and 300 ml undiluted 96% ethyl alcohol (CentralChem, Slovakia)) before being further fixed in 10% neutral formalin liquid. The research was conducted following the permission of the Ethics Committee of Constantine the Philosopher University in Nitra (UKF-2023/1006-2:191013).

### Histological Processing

For histological examination, all 20 samples were processed. The prepared and fixed samples in 10% neutral formalin liquid were rinsed under running water for 1 h. Subsequently, they were cleaned, dehydrated, and impregnated using a descending series of benzyl alcohols (70% and 96%, Slavus, Slovakia), xylene (CentralChem, Slovakia), and paraffin (Shandon Excelsior ES A78410120, Thermo, Germany) for 14 h. The samples were then definitively embedded (Tissue embedding & Cooling system YR442, Klastein France SAS, France) in wax at a temperature of 60 °C and allowed to solidify at room temperature. Histological sections were cut to a final thickness of 3–4.5 μm using a rotary microtome (RM2255, Leica, Germany). The histological sections were stained using the hematoxylin and eosin (H&E) method (Stainer AUS 124, Bio Optica Milano SPA, Italy). They were observed and photographed using a polarized light microscope (Leica DM 750P) at various magnifications.

## Results

### Digestive Tract

**Fig. 1.**
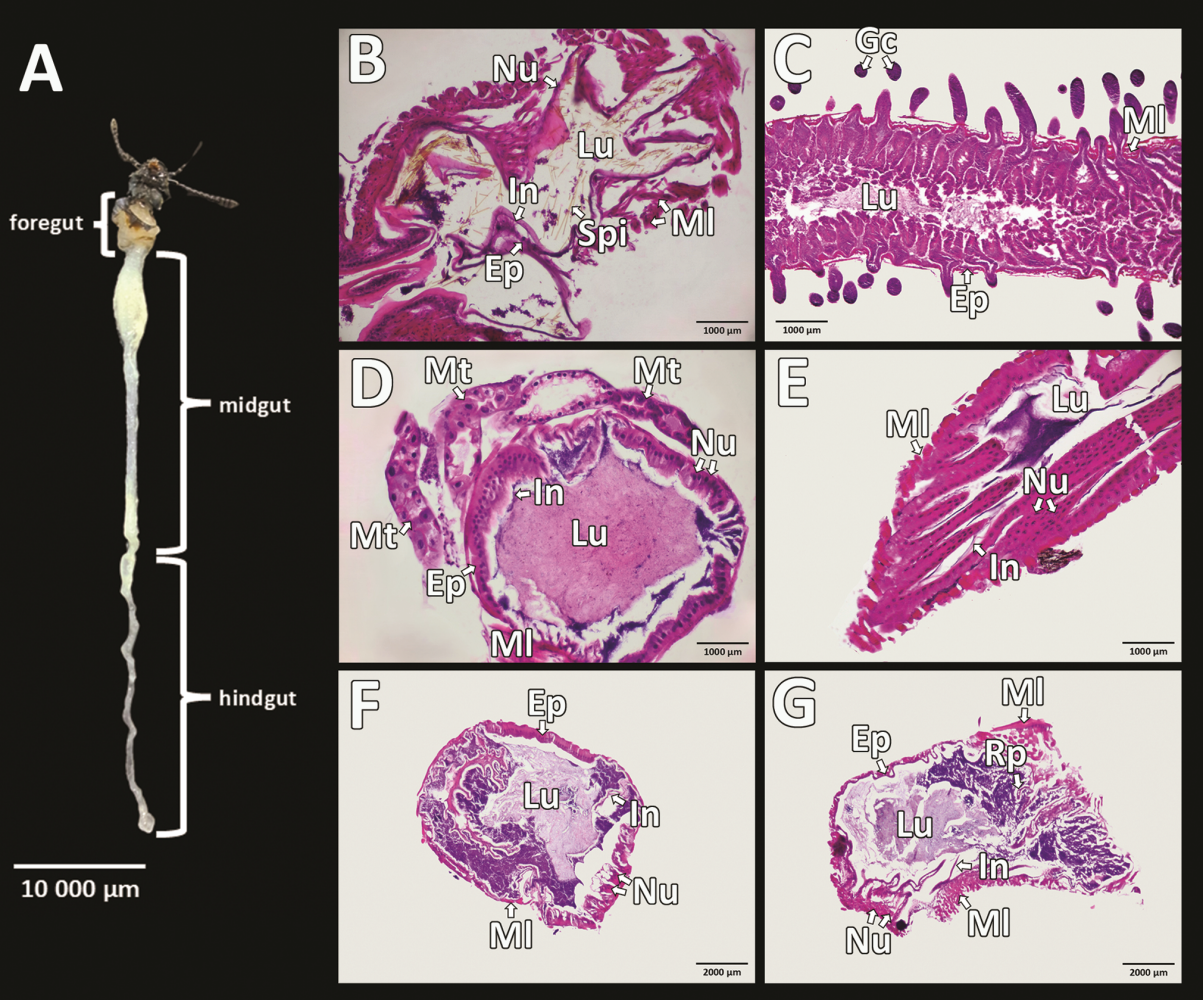
The digestive tract of *Silpha obscura.* A) The foregut, midgut and hindgut; B) Longitudinal section through the foregut; C) Longitudinal section through the midgut along with the crypts of the blind gut; D) Longitudinal section through the Malpighian tubules; E) Longitudinal section through the hindgut—ileum; F) Section through the hindgut—colon; G) Section through the hindgut—rectum. Ep, epithelium; Gc, gastric ceaca; In, cuticle (intima); Lu, lumen; Ml, muscular layer; Mt, Malpighian tubules; Nu, nucleus; Rp, rectal pads; Spi, Spines extending from the intima.

According to the obtained results, the digestive tract of male *S*. *obscura* is relatively straight and has a simple canal. It consists of 3 basic parts: anterior (foregut), middle (midgut), and posterior (hindgut) intestines (Fig. 1A). In the histological analysis of the digestive tract, our focus was on the middle and posterior intestines of the alimentary tract.

### The Foregut

The foregut begins in the oral cavity, housing oral organs. It then continues through the pharynx and esophagus into the expanding proventriculus. The proventriculus contains thorny cuticles within its lumen (Fig. 1B), which aid in food processing (Toni et al. 2022). The foregut is relatively short, narrow at the front, and gradually increases in diameter until it empties into the midgut. The transition between the foregut and midgut is formed by the cardiac valve.

### The Midgut

The midgut is primarily responsible for the digestion and absorption of nutrients (Toni et al. 2022). It consists of a broad anterior part and a narrower posterior tubular part. The wall of the midgut is formed by a single-layered cylindrical epithelium that creates characteristic small folds. This undulating surface allows for an increased area for digestion. The epithelium consists of elongated columnar cells with oval nuclei, which are constantly replaced by new cells produced by specialized regenerative cells. A muscular layer surrounds the midgut; the lumen is wide and filled with secretions and nutrients. The entire surface of the midgut is covered with crypts of the gastric caeca (Fig. 1C). Some of these crypts are in direct contact with the epithelium lining the lumen of the midgut. The crypts of the gastric caeca are surrounded by muscle, beneath which lies a secretory epithelium with visible nuclei and digestive glands. The lumen is narrow and filled with secretions.

### The Hindgut

The midgut and hindgut are separated by the pyloric valve, and this location is characterized by the presence of Malpighian tubules (Fig. 1D), which further extend into the ileum, colon, rectum, and anus. In the beetle *S*. *obscura*, we observed 2 types of Malpighian tubules: one with a straight shape, while the other had a twisted and convoluted form. The Malpighian tubules were pale in color and did not prominently protrude from the digestive tract. Histological analysis indicates that the Malpighian tubules comprise cuboidal epithelium with large oval nuclei. Notably, the cells do not closely adhere to each other, but rather, there are wider spaces between them. The lumen is filled with secretion, and a thin layer of connective tissue covers the surface.

The ileum is covered by a well-developed muscular layer (Fig. 1E). The epithelium is cuboidal with clearly visible oval nuclei, forming numerous folds and bordered by a thin cuticle. The topology of the epithelium in the ileum suggests an increased surface area for the absorption of water and ions. The lumen is filled with intestinal contents.

The colon is a straight tube surrounded by a robust muscular layer consisting of longitudinal and circular muscles. Beneath this layer lies a cylindrical epithelium with visible nuclei, onto which the cuticle is attached. The lumen expands and is filled with intestinal content (Fig. 1F).

The last part of the hindgut is the rectum (Fig. 1G), terminating in the anus, which connects to the genital chamber. The anatomical appearance of the rectum is similar to that of the colon. The rectum also possesses a well-developed muscular system composed of longitudinal and circular muscles responsible for expelling feces. Below them is another muscular layer: a single-layered cuboidal epithelium with an overlaying cuticle. The epithelium with the cuticle forms numerous folds towards the lumen, as the rectum has the ability to dilate. We also observed the presence of rectal pads located on the inner wall of the rectum, visibly separated and lined with epithelium (Fig. 1G).

### Reproductive Tract

**Fig. 2.**
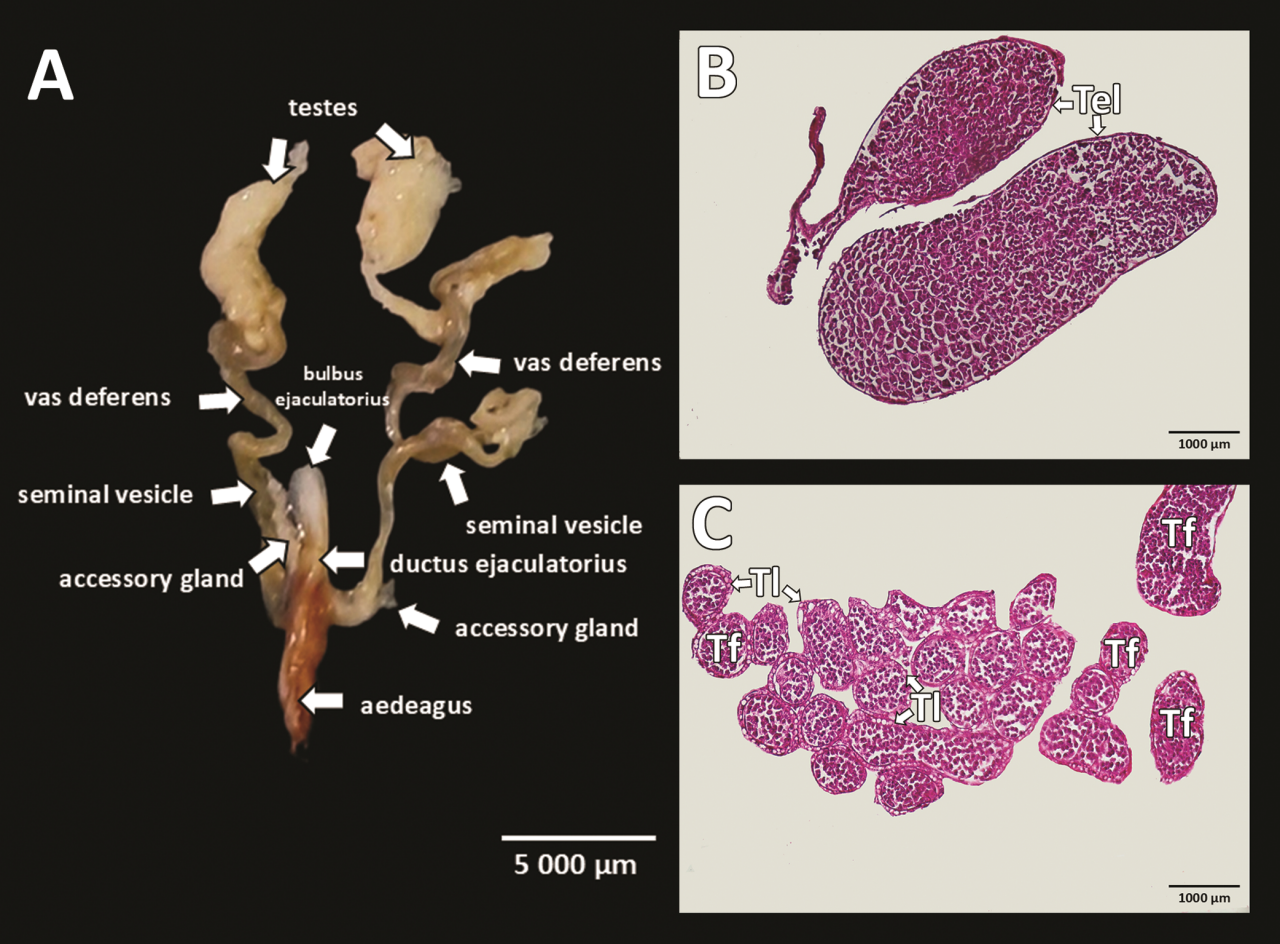
The reproductive system of *Silpha obscura.* A) The anatomy of reproductive system; B) Longitudinal section of the testis; C) Transverse section of the testis. Tel, testis lobe; Tf, testis follicle; Tl, testis tracheoles.

According to the obtained results, we confirm that the reproductive tract of male *S*. *obscura* is similar to that of most Coleoptera species in the suborder Polyphaga. The male reproductive tract of *S*. *obscura* consists of a pair of testes, a pair of efferent ducts (vasa effentia), a pair of deferent ducts (vasa deferentia), 2 pairs of accessory reproductive glands, the ductus ejaculatorius, which extends into the *ejaculatorius bulbus*, and the *aedeagus*. The entire reproductive system exhibits a white-yellowish color (Fig. 2A).

### Testis

**Fig. 3.**
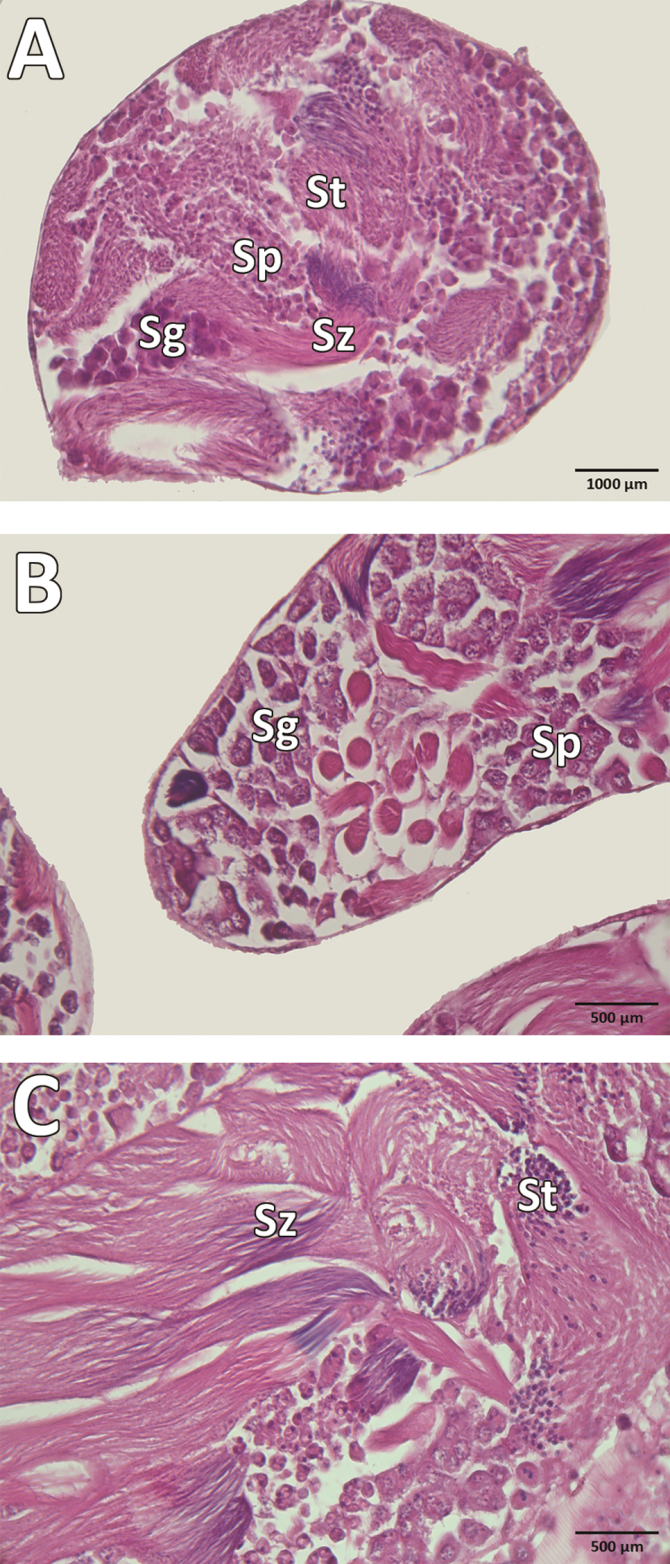
The testis follicle of *Silpha obscura.* A) Transverse section through the testis follicle; B and C) Longitudinal section through the testis follicle. Sg, spermatogonia; Sp, spermatocysts; St, spermatids; Sz, spermatozoa.

**Fig. 4.**
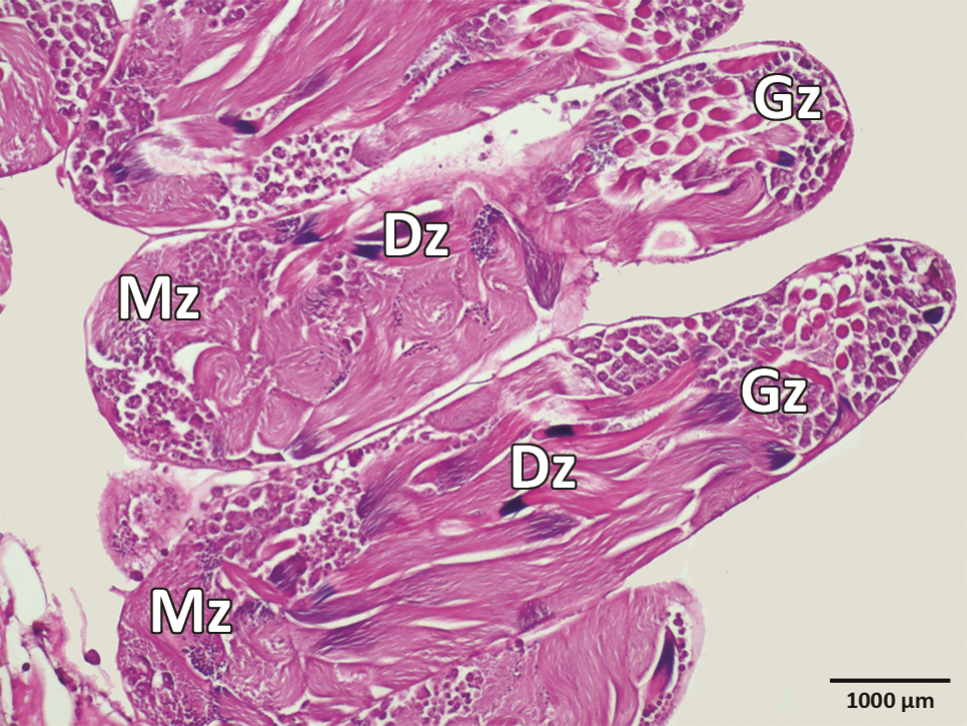
Developmental zones of spermatogenesis and spermiogenesis. Gz, growth zone; Dz, differentiation zone; Mz, maturation zone.

The testes are whitish in color, located on the lateral sides of the abdominal cavity, elliptical in shape, composed of 2 lobes, and folded from numerous follicles. Bundles of follicles create a broccoli-like appearance. Each lobe is surrounded by a membranous envelope carrying a network of tracheoles (Fig. 2B and C). Spermatogenesis occurs in each testicular follicle. Our results indicate that in *S*. *obscura*, the development proceeds as follows: spermatogonia, spermatocytes, spermatids, and spermatozoa (Fig. 3). The development of sperm progresses from the peripheral part of the follicle to the center. In the growth zone, spermatogonia undergo mitotic divisions and differentiate into spermatocytes. Numerous spermatocytes are clustered in the follicle, with aggregated heads visible. In the maturation zone, spermatocytes undergo meiosis and differentiate into spermatids whose head and tail are distinguishable. In the differentiation zone, spermatids differentiate into spermatozoa arranged in regular bundles (Figs. 3 and 4).

### Vas Deferens

**Fig. 5.**
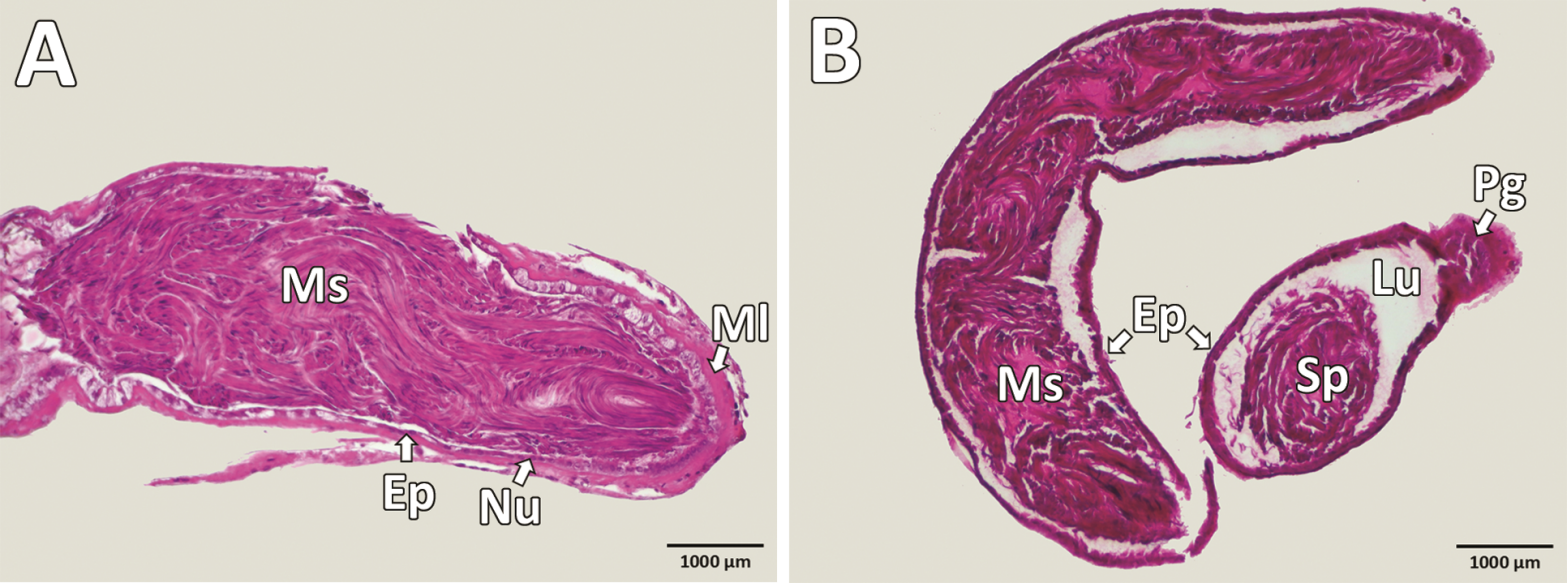
Vas deferens in the beetle *Silpha obscura.* A) Longitudinal section; B) Transverse and longitudinal section through the vas deferens and accessory prostatic gland. Ep, epithelium; Lu, lumen; Ml, muscle; Ms, mature sperm; Nu, nucleus; Pg, prostatic gland; Ms, mature sperm.

From each lobe of the testis, a thin and delicate duct (vas efferens) emerges, connecting the testis to the vas deferens. The vas deferens are relatively thin, long paired tubular structures with various twists in the anterior part (near the testis). Subsequently, they straighten out and smoothly transition into the seminal vesicles. The seminal vesicles form an expanded section of the vas deferens and serve as a reservoir for sperm. Mature sperm then travel through the vas deferens into the seminal vesicles. The vas deferens are covered by a muscular layer that facilitates peristaltic movements, allowing smooth passage of sperm into the ejaculatory duct. Beneath this layer is a single-layered cylindrical epithelium with clearly visible nuclei. The entire lumen of the vas deferens is filled with sperm (Fig. 5A).

### Accessory Glands

**Fig. 6.**
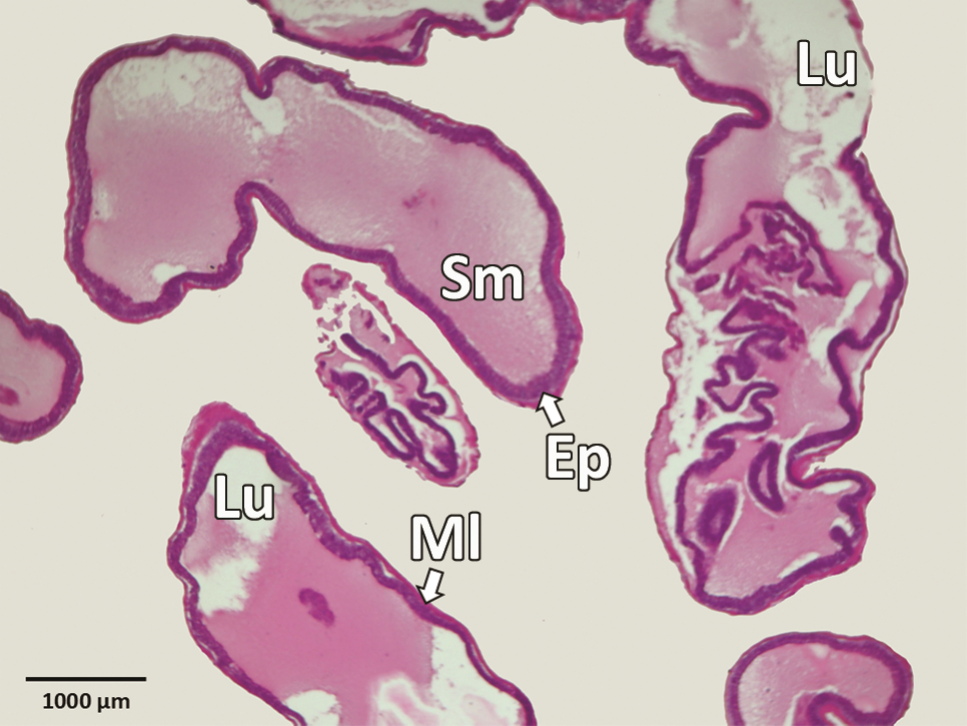
Longitudinal section through the accessory reproductive glands of *Silpha obscura.* Ep, epithelium; Lu, lumen; Sm, secretory material.

In close proximity to the vas deferens are the accessory reproductive glands, which are taxonomically specific. In *S*. *obscura*, the prostate glands are single-lobed and adhere to the vas deferens (Fig. 5B). They are surrounded by a single-layered cylindrical epithelium containing secretory cells. The second type consists of long, tubular, and coiled accessory reproductive glands. These glands have a single-layered cuboidal epithelium (Fig. 6). The lumen of the glands is relatively large and filled with secretory fluids. Accessory reproductive glands occur in pairs and function to form seminal fluid, which protects sperm and facilitates their transfer. Additionally, these glands contribute to the formation of a spermatophore.

### Ductus Ejaculatorius

**Fig. 7.**
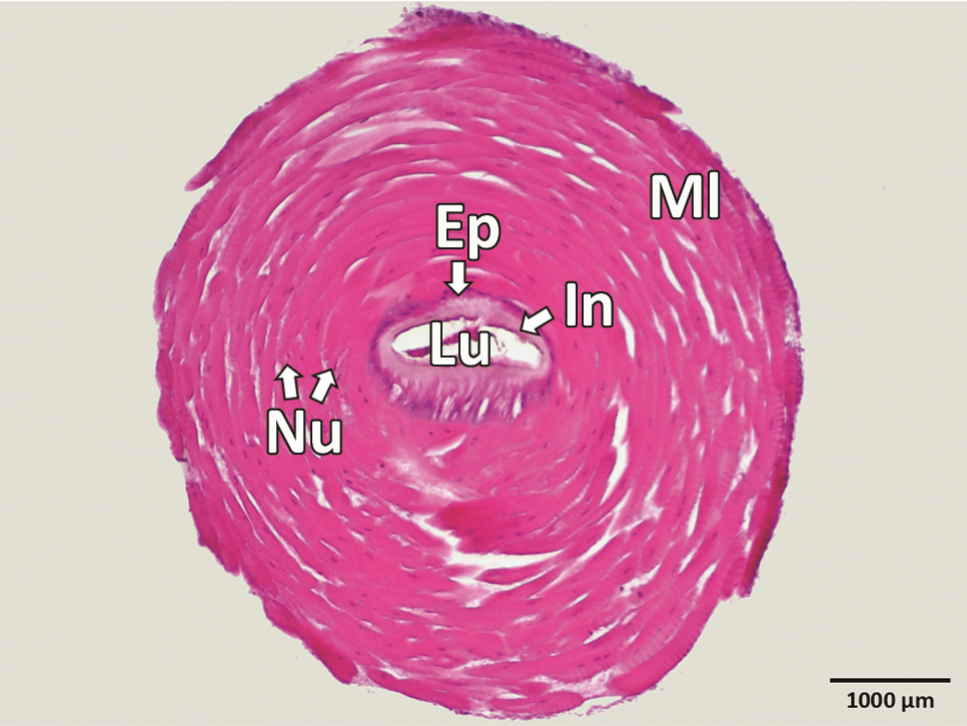
Transverse section of the ejaculatory duct (*ductus ejaculatorius*) in *Silpha obscura.* Ep, epithelium; In, intima (cuticle); Lu, lumen; Ml, muscle; Nu, nucleusZačiatok formulára.

The vas deferens and the accessory reproductive glands converge into the ductus ejaculatorius. In *S*. *obscura*, the ductus ejaculatorius is expanded in the upper part, forming the ejaculatory bulb (*bulbus ejaculatorius*; Fig. 7). The most significant and dominant portion is the thick-walled muscular covering, which consists of robust longitudinal muscles with clearly visible nuclei, facilitating controlled contraction for the transport of secretions through the aedeagus into the female’s body. Beneath the muscular layer is a single-layered epithelium covered with cuticle. During ejaculation, sperm, along with secretory fluids, are transported through the lumen into the female’s body.

The terminal part of the ductus ejaculatorius is the sclerotized aedeagus, which is covered with a cuticle.

## Discussion

### Digestive Tract

The anatomical and histological structure of the digestive tract in *S*. *obscura* shows notable similarities and differences compared with other beetles, particularly within the Silphidae family (Candan et al. 2019, 2021, Özyurt Koçakoğlu et al. 2022, Toni et al. 2022). Our findings revealed that *S*. *obscura* shares a generally similar digestive tract morphology with *Oxelytrum discicolle* (Silphidae) (Toni et al. 2022), including the elongated midgut and extensive gastric caeca crypts. However, differences were observed in the arrangement and density of these crypts, which were uniformly distributed across the entire midgut in *S*. *obscura*, potentially enhancing digestive efficiency in response to its omnivorous diet (Candan et al. 2019, 2021, Özyurt Koçakoğlu et al. 2021a, 2021b, Toni et al. 2022).

Previous studies on the Silphidae, such as *Nicrophorus vespilloides* (Nicrophorinae) (Vogel et al. 2017), documented pouch-shaped crypts along the midgut, contrasting with the widespread crypts observed in *S. obscura*. This suggests that the arrangement of gastric caeca may be more variable within the Silphidae than previously thought, possibly reflecting dietary adaptations. The uniform distribution of crypts in *S*. *obscura* may provide a larger surface area for nutrient absorption, potentially an adaptive advantage for omnivorous feeding.

The histological structure of the midgut, characterized by a single-layered cylindrical epithelium with columnar cells and regenerative crypts, aligns with observations in other Coleoptera, including *Calosoma sycophanta* (Carabidae) (Candan et al. 2021) and *Mylabris cernyi* (Özyurt Koçakoğlu et al. 2022). However, the regenerative crypts in *S*. *obscura* are more directly associated with the lumen lining, possibly indicating a higher turnover rate of epithelial cells to support continuous digestion. This feature may represent a specialized adaptation within the Silphidae, potentially linked to their ecological role as decomposers and scavengers.

Regarding the Malpighian tubules, the structure and arrangement in *S*. *obscura* showed similarities with *Melanophila picta decastigma* (Buprestidae) (Özyurt Koçakoğlu et al. 2021b), where 3 pairs of tubules are observed. However, the presence of a light-colored appearance, as opposed to the diverse colors noted in other beetles (Candan et al. 2021), suggests a different composition of secretory substances or varying levels of waste products. The relatively simpler structure could indicate less specialization in excretory function compared to beetles with more complex diets or habitats.

The hindgut of *S*. *obscura* shares a common composition with many Coleoptera, including a cylindrical epithelium with attached cuticle and muscular layers. However, the district, elongated rectum with numerous folds aligns closely with *O*. *discicolle* (Silphidae) (Toni et al. 2022). The well-developed muscle system observed in *S*. *obscura* supports efficient waste expulsion, which is consistent with findings in other beetles, such as *Carabus violaceus* (Carabidae) (Candan et al. 2021).

The ileum is a narrow-walled tube located between the pyloric valve and the colon, with a well-developed muscular layer. Two types of muscles, longitudinal and transverse, are described in *C*. *sycophanta* (Carabidae) (Candan et al. 2021), facilitating necessary peristalsis. The epithelium of the ileum is cuboidal with clearly visible oval nuclei, forming numerous folds and lined with a thin cuticle. Similar structures are visible in *Platynotus belli* (Tenebrionidae) (Sarwade and Bhawane, 2013), *Epiphaneus malachiticus* (Curculionidae) (Candan et al. 2019), *Chrysomela populi* (Chrysomelidae) (Özyurt Koçakoğlu et al. 2021a), *M*. *cernyi* (Meloidae) (Özyurt Koçakoğlu et al. 2022), *M*. *picta decastigma* (Buprestidae) (Özyurt Koçakoğlu et al. 2021b), and *O*. *discicolle* (Silphidae) (Toni et al. 2022). The complex epithelium in the ileum increases the area for water and ion absorption, a function common in Coleoptera and also observed in *Passalus* (*Pertinax*) *punctatostriatus* (Passalidae) (González-Gómez et al. 2010), *Capnodis tenebrionis* (Buprestidae) (Özyurt Koçakoğlu et al. 2020), and *O*. *discicolle* (Silphidae) (Toni et al. 2022).

The colon in *S*. *obscura* has the form of a straight tube, similar to *M*. *cernyi* (Meloidae) (Özyurt Koçakoğlu et al. 2022), unlike *M*. *picta decastigma* (Buprestidae) (Özyurt Koçakoğlu 2021b), which forms a star-shaped cross-section of the lumen. It is covered by a muscular layer consisting of longitudinal and transverse muscles, similar to *C*. *sycophanta* (Carabidae) (Candan et al. 2021) and *M*. *picta decastigma* (Buprestidae) (Özyurt Koçakoğlu 2021b). Beneath this layer is a cylindrical epithelium with visible nuclei to which the cuticle is attached.

The last part of the hindgut is the rectum, which connects the ileum to the anus. The digestive tract joins the aedeagus in the genital chamber at the end (Özyurt Koçakoğlu et al. 2022). The rectum exhibits a well-developed muscular system composed of longitudinal and circular muscles, facilitating the expulsion of fecal matter (Borges et al. 2015, Özyurt Koçakoğlu et al. 2021a, 2022, Toni et al. 2022). The rectal epithelium is a single-layered cuboidal epithelium onto which the cuticle is attached. The epithelium with cuticle forms numerous folds toward the lumen, as observed in *C*. *populi* (Chrysomelidae) (Özyurt Koçakoğlu et al. 2021a), *C*. *sycophanta* (Carabidae) (Candan et al. 2021), *E*. *malachiticus* (Curculionidae) (Candan et al. 2019), and *P*. *belli* (Tenebrionidae) (Sarwade and Bhawane 2013).

In all terrestrial insects, rectal pads are present at the anterior end of the rectum. These structures resemble an inverted bowl, similar to the rectal gland, with cells reabsorb water from the excrement (Haridass and Ananthakrishnan 1981, Özyurt Koçakoğlu et al. 2021b). Rectal pads were observed on the inner wall of the rectum, visibly separated and lined with epithelium, similar to *C*. *sycophanta* (Carabidae) (Candan et al. 2021) and *M*. *picta decastigma* (Buprestidae) (Özyurt Koçakoğlu et al. 2021b). However, they were not documented in *O*. *discicolle* (Silphidae) (Toni et al. 2022).

### Reproductive Tract

Morphological characteristics of the male reproductive tract in insects, particularly the structure of the testes and accessory glands, are valuable for taxonomic and phylogenetic studies (Dallai 2014, Salazar et al. 2016, Özyurt Koçakoğlu et al. 2019). In *S*. *obscura*, our findings provide a detailed description of the reproductive tract that not only aligns with some known patterns but also presents distinct traits not previously reported in other species.

In the case of multicellular follicles observed in *S*. *obscura*, the overall appearance of the testes resembles a “broccoli head,” comparable to the testes of *Pimelia subglobosa* (Tenebrionidae) (Özyurt Koçakoğlu and Candan 2022). The color of the testes is often inconspicuous and whitish, as indicated by our findings. However, there are individuals with much more pronounced colors, such as *C*. *tenebrionis* (Buprestidae) (Çağlar et al. 2020) or *Isotomus speciosus* (Cerambycidae) (Özyurt Koçakoğlu et al. 2023), which exhibit a vivid yellow hue in their testes.

On the surface of the testis in *S*. *obscura*, we observed an epithelium containing a network of tracheoles. This tracheolar network has also been observed in *C*. *tenebrionis* (Buprestidae) (Çağlar et al. 2020) and *Phyllobius* (*Ectomogaster*) *fulvago* (Curculionidae) (Erbey et al. 2021), among many others.

The pattern of spermatogenesis and spermiogenesis in *S*. *obscura* aligns with that observed in various Curculionidae (Özyurt Koçakoğlu et al. 2019, Erbey et al. 2021), where development proceeds from the germinal cell to mature spermatozoa. In testicular follicles, there are 3 distinct zones: growth, maturation, and differentiation. However, the follicular structure in *S*. *obscura* appears to support a more homogenous distribution of germ cells (cysts) at various developmental stages, differing from the more compartmentalized arrangement noted in species with elongated testes (Özyurt Koçakoğlu et al. 2023). This suggests that testicular shape may influence germ cell organization and spermatogenesis efficiency.

In the center of each testicular lobe, there is a thin vas efferens that connects each lobe to paired vasa deferentia, as observed in *I*. *speciosus* (Cerambycidae) (Özyurt Koçakoğlu et al. 2023), *Spasalus silvarum* (Passalidae) (Salazar et al. 2016), *C*. *tenebrionis* (Buprestidae) (Çağlar et al. 2020), and *P*. *fulvago* (Curculionidae) (Erbey et al. 2021).

The vasa deferentia are thin, long tubes of mesodermal origin that serve as the excretory ducts of the testes, with various twists or dilations. Vasa deferentia are covered with a muscular layer responsible for peristalsis. In *S*. *obscura*, the vas deferens is covered with a single-layered cylindrical epithelium with visible nuclei, similar to *C*. *tenebrionis* (Buprestidae) (Çağlar et al. 2020). The entire lumen is filled with sperm.

The accessory glands are in close proximity to the sperm ducts. The prostatic glands in *S*. *obscura* are single-lobed, differing from the multiple-lobed structures in *Trypophloeus klimeschi* (Curculionidae) (Gao et al. 2021) and the tubular accessory glands in *C*. *tenebrionis* (Buprestidae) (Çağlar et al., 2020). This variation in glandular structure implies functional differentiation among species, possibly related to seminal fluid composition and its role in sperm activation and transfer.

The direction of sperm development depends on the shape of the testis. If the testes are round, sperm development occurs from the peripheral region towards the center, as seen in *C*. *tenebriunis* (Buprestidae) (Çağlar et al. 2020) and *Lasioderma serricorne* (Ptinidae) (Reis et al. 2021), as well as in our observations of *S*. *obscura*. If the testes are elongated, the direction of sperm development is from the distal part to the proximal region (Özyurt Koçakoğlu et al. 2023), as observed in *P*. *fulvago* (Curculionidae) (Erbey et al. 2021), *I*. *speciosus* (Cerambycidae) (Özyurt Koçakoğlu et al. 2023), and *Pimelia subglobosa* (Tenebrionidae) (Özyurt Koçakoğlu and Candan, 2022).

The bulbous expansion in the upper part of the ejaculatory duct in *S*. *obscura* is similar to structures observed in certain Pentatomidae species (Özyurt et al. 2015), but it is not universally present across all beetles. For example, *Gammarus lacustris* (Candan et al. 2018) lacks this feature. This difference may affect the mechanics of sperm transfer and the formation of the spermatophore.

The terminal part of the ejaculatory duct is sclerotized, forming the aedeagus. The aedeagus is covered with a cuticle and is concealed in the genital chamber, distinct from a pair of ectodermal lobes associated with the 9th abdominal segment (Karakaya et al. 2012, Özyurt et al. 2013, 2015).

## Conclusion

The digestive and reproductive tracts of insects have relatively complex structures that vary from species to species. Examining the anatomical and histological structure of these tracts is crucial for managing these pests. Therefore, in this study, we focused on describing the digestive and male reproductive tract of *S. obscura* and highlighted its similarities and differences with other species. Our aim was to contribute to the understanding of these aspects.
